# Gas sensing with gold-decorated vertically aligned carbon nanotubes

**DOI:** 10.3762/bjnano.5.104

**Published:** 2014-06-26

**Authors:** Prasantha R Mudimela, Mattia Scardamaglia, Oriol González-León, Nicolas Reckinger, Rony Snyders, Eduard Llobet, Carla Bittencourt, Jean-François Colomer

**Affiliations:** 1 Research group on Carbon Nanostructures (CARBONNAGe), University of Namur, Belgium; 2 Chimie des Interactions Plasma-Surface (ChIPS), CIRMAP, Research Institute for Materials Science and Engineering, University of Mons, Mons, Belgium; 3 MINOS-EMaS, Universitat Rovira i Virgili, Tarragona, Spain

**Keywords:** alignment, carbon nanotubes, decoration, gas sensors, metal nanoparticles, thermal CVD

## Abstract

Vertically aligned carbon nanotubes of different lengths (150, 300, 500 µm) synthesized by thermal chemical vapor deposition and decorated with gold nanoparticles were investigated as gas sensitive materials for detecting nitrogen dioxide (NO_2_) at room temperature. Gold nanoparticles of about 6 nm in diameter were sputtered on the top surface of the carbon nanotube forests to enhance the sensitivity to the pollutant gas. We showed that the sensing response to nitrogen dioxide depends on the nanotube length. The optimum was found to be 300 µm for getting the higher response. When the background humidity level was changed from dry to 50% relative humidity, an increase in the response to NO_2_ was observed for all the sensors, regardless of the nanotube length.

## Introduction

The interest in gas sensing for reaching a widespread, continuous pollution detection and control has been growing steadily in the last decades due to the increasing impact in our environment of human activities. The detection of typical pollutants such as nitrogen dioxide (NO_2_) generated by industrial combustions or by car emissions is critical because of both environmental problems and health consequences for humans. In the last few years, new technologies based on nanomaterials have been developed to fabricate small and inexpensive gas sensors with high sensitivity and able to work at room temperature [1]. Among the possible active materials in gas sensing devices, good candidates are carbon nanotubes (CNTs), thanks to their intrinsic properties such as very large surface area to volume ratio, high electron mobility, physico-chemical stability and high adsorption capability [2–5]. The use of CNTs as gas sensors was first proposed by Kong et al., who showed that a dramatic change in the electrical resistance of an individual single-walled nanotube occurs upon exposure to different gases, including NO_2_ [5]. Since this first study, gas sensors based on CNT films, i.e., random CNT networks (single- or multi-walled nanotubes) were intensively investigated and shown to exhibit, in some particular cases, high sensitivity (even when operated at room temperature), prompt response, short recovery time and reasonable reversibility and stability [2–4,6]. A further advance in the development of CNT gas sensing devices was the use of vertically aligned CNTs (VA-CNTs). In this case the sensing device benefits from the unidirectional electrical charge transport [7], unlike in randomly oriented CNT meshes. Sensors made of aligned CNTs synthesized by plasma enhanced chemical vapour deposition (PECVD), have been reported to exhibit fast and high response at room temperature and detect 10 ppb NO_2_ when operated at 165 °C. Although the detection of 10 ppb of nitrogen dioxide is possible at room temperature, the sensor does not recover its baseline resistance when cleaning in dry air at such low temperature [8]. Ueda et al. using VA-CNTs synthesized by thermal CVD as sensing active layer showed that variation in the conductivity was proportional to the concentration of target gas, such as NO_2_ [9]. However, the low chemical reactivity and selectivity of the pristine CNTs associated to their structure consisting of a hexagonal network of sp^2^-hybridized C atoms had prevented their use as sensing layer. Consequently, the functionalization of CNTs has been reported as a good strategy to enhance the sensitivity and the selectivity of the CNT-based sensors [10–12]. The conception of CNT-metal cluster hybrids has been developed as the sensitive material of the device, where the metal cluster surfaces act as reactive sites for the adsorption of the target molecules [13]. This was firstly shown using palladium on individual single-walled nanotubes in the detection of H_2_ with a 50% higher response than the sensor made of pristine carbon nanotubes [14]. Following this report, it was showed that the gas sensitivity of metal (Au, Pt)-functionalized multi-walled nanotubes randomly arranged was significantly improved for NO_2_ gas detection [15]. Later, VA-CNTs produced by PECVD were functionalized with nominally 5 nm-thick metal nanoparticles by magnetron sputtering, providing a higher sensitivity to NO_2_ [16]. Based on these reported results, it was shown that the sensitivity of the CNT gas sensor depends on nanocluster size and sensor working temperature [13–17]. Indeed, the nanoscale size of the metal cluster is necessary to maximize the effect of the gas adsorption and so to affect the electron transport in the CNTs by charge transfer. Concerning the nature of metal nanocluster, it has been shown that despite the fact that gold is inert in bulk form, it is catalytically active in the nanometer range [18]. This increased reactivity has been explained by the presence of low-coordinated gold atoms such as the atoms on the corners and edges of particles, particularly abundant on the nanometer-sized particles [19–20]. Accordingly, the loading of gold nanoparticles has been reported to strongly influence CNT sensor sensitivity [17,21–22].

In this work we study the influence of the length of aligned CNTs decorated with gold particles in the gas sensing applications. We investigated the sensing response of VA-CNTs with different lengths (150, 300 and 500 µm), decorated with gold nanoparticles, to a NO_2_ atmosphere at room temperature. Moreover, the effect of humidity from dry to 50% R.H. on the gas sensing was also studied. The morphology of the active layer and its chemical composition was characterized by using scanning and transmission electron microscopies (SEM and TEM), and X-ray photoelectron spectroscopy (XPS), respectively.

## Experimental

### Vertically aligned carbon nanotube growth

VA-CNT synthesis was carried out in a thermal CVD reactor using C_2_H_4_ as carbon source. Si wafers with native SiO_2_ were used as substrates. Al_2_O_3_ (30 nm) were used as buffer layer on wafer pieces and Fe (6 nm) used as active catalyst. The multilayer system composed of Si/native SiO_2_/Al_2_O_3_/Fe will be called from now the catalyst. The Al_2_O_3_ and Fe layers were deposited under 2 mTorr and 20 mTorr pressures using radio frequency (RF) and direct current (DC) magnetron sputtering, respectively. For the CNT growth, the reactor was heated to 750 °C at atmospheric pressure under Ar flow (120 sccm). The catalyst was placed inside the reactor and the H_2_ flow (120 sccm) was introduced into the reactor. After 5 min, Ar was replaced by the C_2_H_4_ flow (50 sccm). The growth time was varied from 7 to 30 min for synthesizing VA-CNTs with different lengths (150, 300 and 500 µm). After the growth, H_2_ and C_2_H_4_ were replaced by Ar (120 sccm). Finally, the sample was taken out from the reactor. The detailed synthesis was reported in [23].

### Formation of gold nanoparticles

Gold nanoparticles were prepared by the physical vapor deposition (PVD) technique. This method is well-known to synthesize nanoparticles based on the aggregation of free atoms generated in the gas phase by DC magnetron sputtering (with a power of 75 W) from a gold target under Ar atmosphere (180 mTorr). It allows the production of well-dispersed nanoparticles in the gas phase with a narrow size distribution, production independent of the target substrate. The gold nanoparticles synthesized have a mean diameter of 6 ± 2 nm [24].

### Material characterization

The alignment and the length of the VA-CNT samples were investigated using field emission SEM (FE-SEM) on a JEOL 7500F microscope. TEM was used to determine the metal cluster size and their localization on the CNTs. For that, a part of the VA-CNTs was removed from the surface and dispersed in ethanol, and a drop of this solution was deposited onto a commercial lacey-carbon grid. The TEM experiments were carried out with a FEI Tecnai 10 and a Philips CM200 for higher resolution, operating at 80 and 200 kV, respectively. Raman spectroscopy was performed at room temperature with a LabRam Horiba spectrometer with a laser wavelength of 514 nm.

In order to evaluate the chemical composition of the samples’ surface, XPS was performed in a VERSAPROBE PHI 5000 from Physical Electronics, equipped with a monochromatic Al Kα X-ray source with a highly focused beam size set at 200 μm. The energy resolution was 0.7 eV. The binding energies were calibrated using the Au 4*f* peaks.

### Sensor preparation

Parallel silver electrodes, with an electrode gap of 800 µm, were drop casted on top of the VA-CNTs samples. A commercial Ag paste (Heraeus AD 1688-06) was employed. The electrodes were cured at 170 °C for 30 min. The backside of the samples was glued with a thermally conductive epoxy to an alumina support which had a screen printed Pt resistor to be used as a heater. Au wires were employed to connect both the electrodes and the heater resistor to a small PCB board that can be plugged into our sensor test chamber.

### Measurement set-up

In a typical measurement cycle, dry air was flowed through the test chamber for at least 30 min for allowing the sample resistance to stabilize (i.e., a stable baseline is reached). Then a given concentration of nitrogen dioxide was introduced into the test chamber and kept constant for 15 min. This was implemented by mixing the flows of dry air and nitrogen dioxide diluted in dry air. The total flow remained always constant and equal to 100 sccm during the detection of NO_2_ and cleaning phases. The measurement rig comprises a set of computer-controlled mass flow meters and electro-valves to ensure that reproducible concentrations of the gases are delivered to the test chamber. During detection, the samples were kept at 30 °C. At the cleaning phase, the flow was switched again to dry air and heating was applied to the sample (150 °C) for about 30 min. During the whole process of response and recovery, an AGILENT multimeter was employed to acquire the DC resistance of the sensors. When measurements were performed in the presence of humidity, the flow of NO_2_ diluted in dry air was humidified using a liquid mass flow system.

## Results and Discussion

### Morphology and structure characterization of gold-decorated VA-CNTs

**Figure 1:**
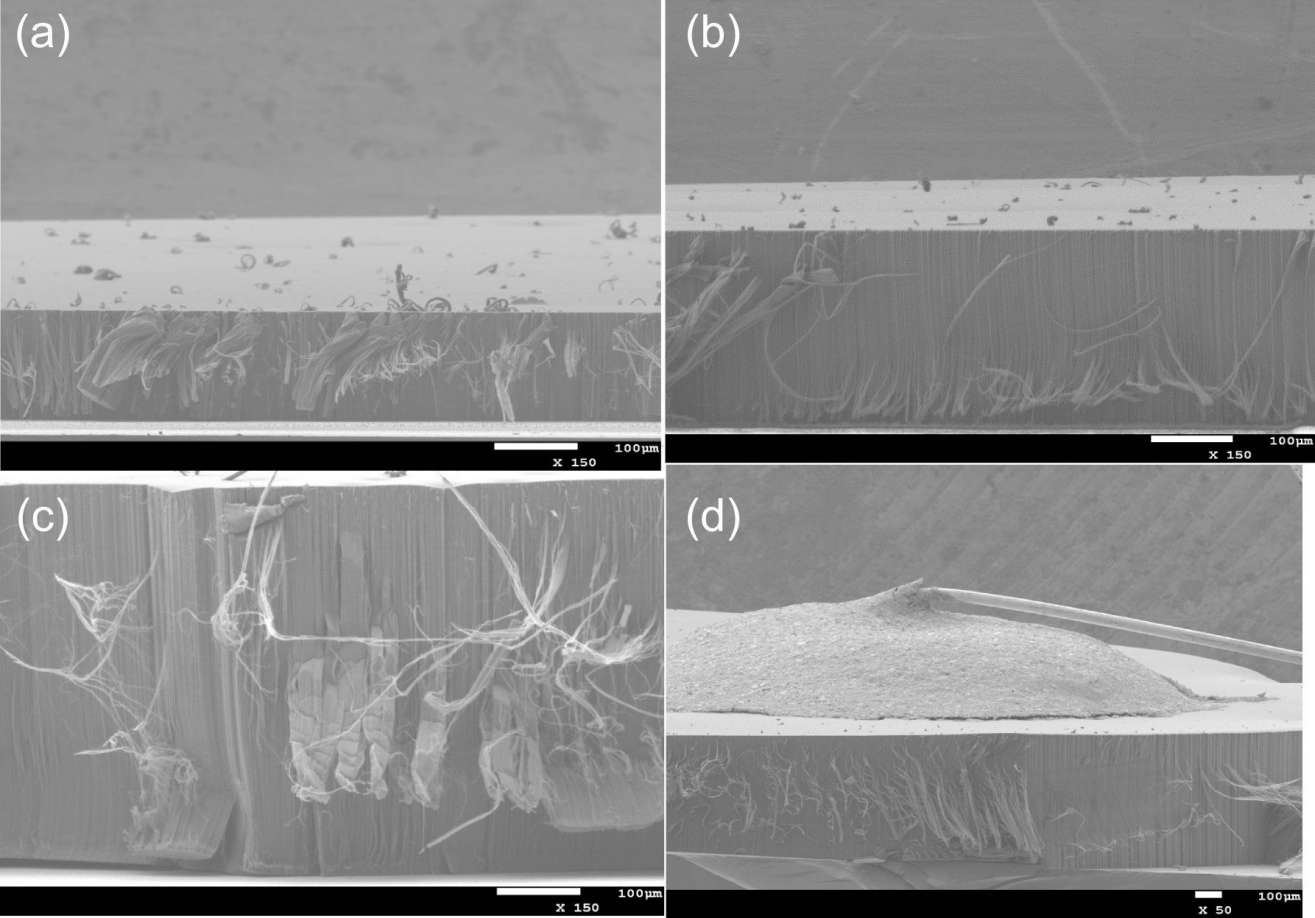
FE-SEM images of sensor devices made of VA-CNTs decorated with gold nanoparticles with different CNT lengths of (a) 150 µm, (b) 300 µm, (c) 510 µm and (d) image of a silver electrode drop casted on top of the device.

Before investigating the sensing response of VA-CNTs decorated with gold nanoparticles to the detection of NO_2_ gas, the morphology of the active layers and their chemical composition are characterized by using SEM, TEM, and XPS. The FE-SEM images present VA-CNT rugs with different lengths used in the experiments (Figure 1). The length of the CNTs increases linearly with growth time at the beginning and the growth rate decreases after a certain time (depending on the experimental conditions) due to the poisoning of the catalysts [23]. The CNT growths were realized with three different times: 7, 15 and 30 min, giving rug lengths of 150, 300, and approximately 500 µm (Figure 1a, b and c, respectively). The growth rate in the CVD conditions used in this study was estimated to be 17.5 µm/min. The last image (Figure 1d) illustrates, for a sample with a rug of about 500 µm-long tubes, the silver electrode drop casted on top of VA-CNTs.

**Figure 2:**
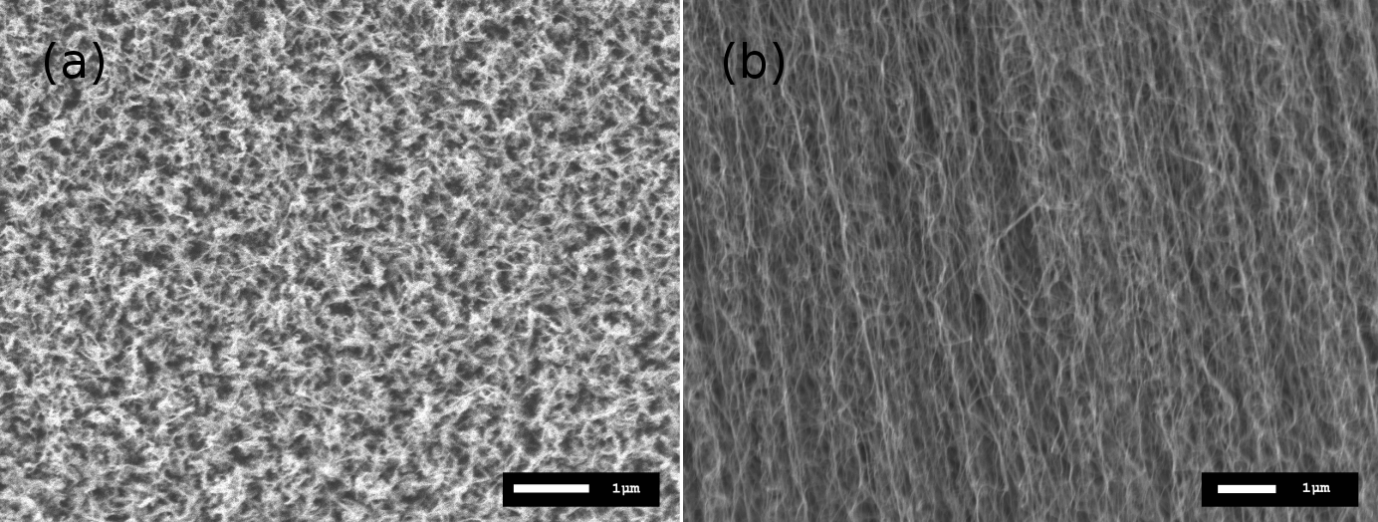
(a) Top and (b) side SEM views of the VA-CNTs.

The SEM images in Figure 2 are shown to exemplify the general morphology of the sensor. The top-view is similar to that of non-aligned CNTs where they seem randomly oriented (Figure 2a). Note that the gold nanoparticles were difficult to observe on top of VA-CNTs due to their diameters. The side view shows the alignment direction of the CNTs, perpendicularly to the substrate surface (Figure 2b).

**Figure 3:**
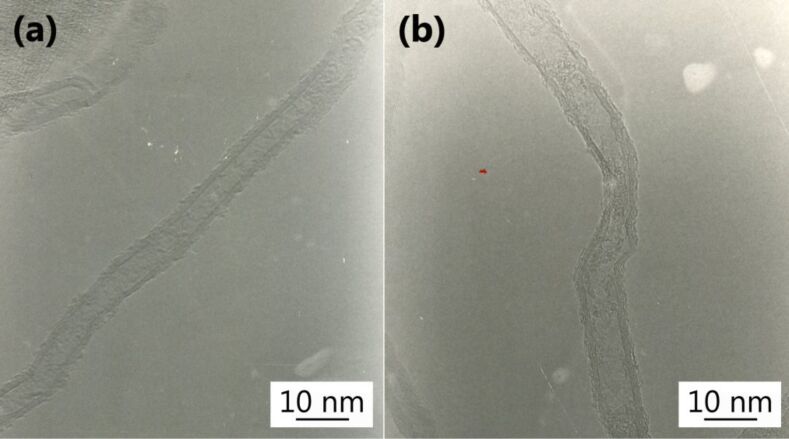
High resolution TEM images of the carbon nanotubes constituting the forests.

The high resolution TEM images show the details of the nanotubes aligned inside the forest (Figure 3). The CNTs were found to consist of multi-walled nanotubes with different wall number (the average value was about a dozen walls). Moreover, the nanotubes showed structural defects as exemplified in Figure 3b. The presence of defects along the length of the tube is related to the relatively low synthesis temperature used (750 °C) [25]. These defects are generally imperfections along the graphitic walls (breaks, dangling bonds due to the presence of sp^3^ carbon, etc.) and explained the low crystallinity of the CNTs [25].

**Figure 4:**
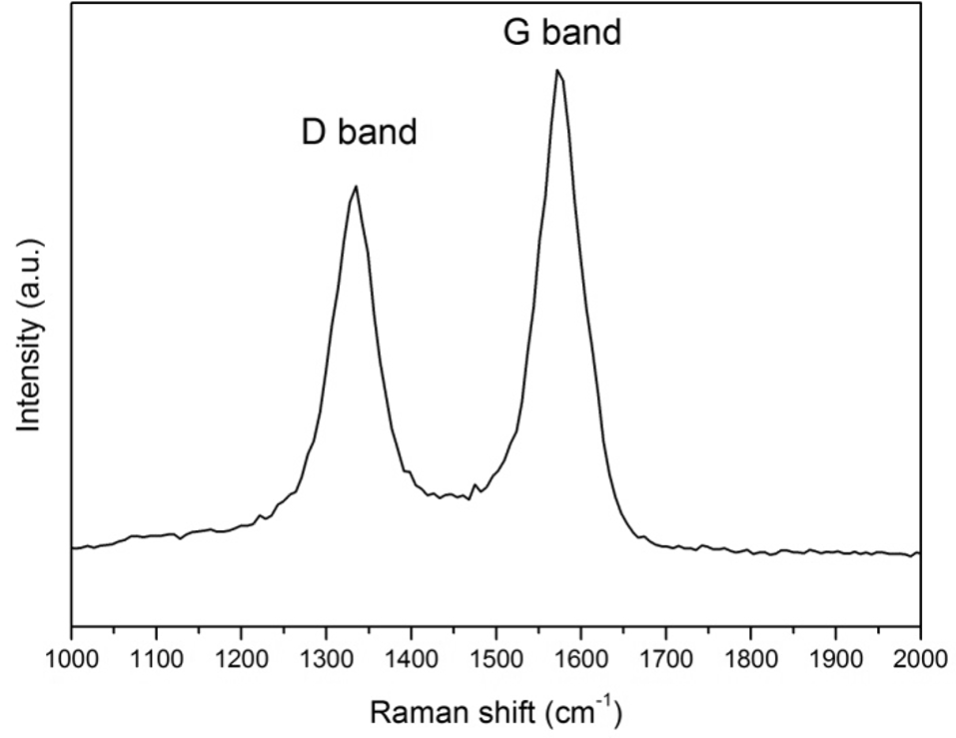
Typical Raman spectrum of VA-CNT sensors.

Raman spectroscopy was used to compare the density of defects for the different samples. Figure 4 shows the typical Raman spectrum recorded on the studied samples. This figure shows the first-order characteristic peaks with a G band at 1575 cm^−1^ related to the in-plane oscillations of sp^2^ carbon atoms due to the graphitic nature of the nanotubes [26] and a D band peak at 1332 cm^−1^ indicating the presence of defects (carbonaceous impurities, broken sp^2^ bonds in the sidewalls, etc.) [27]. The intensity ratio D/G bands was similar for all samples. The presence of a high intensity D band in the Raman spectrum supports the conclusion drawn from the high resolution TEM images (Figure 3), because its high intensity indicates the low crystallinity of the VA-CNTs.

**Figure 5:**
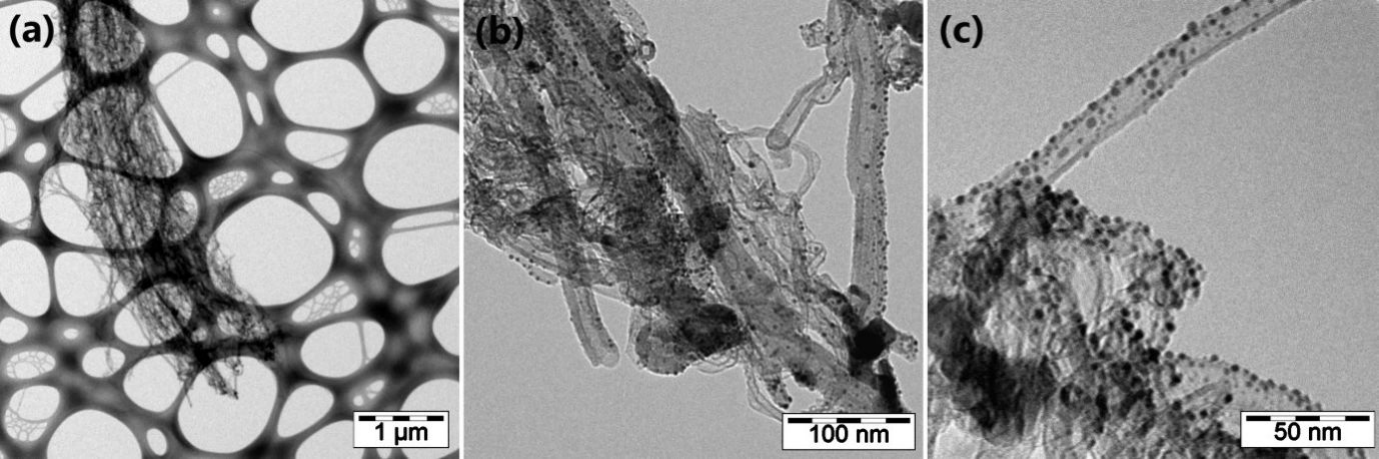
TEM images of VA-CNTs decorated with gold nanoparticles.

After characterizing the CNTs, the gold nanoparticles on top of the nanotube forest were investigated using TEM in order to evaluate their distribution at the surface of the CNTs (Figure 5). After the dispersion of the VA-CNTs in ethanol, a drop of the solution was deposited onto a commercial lacey-carbon grid which is well-visible in Figure 5a. Despite the dispersion of CNTs for the characterization, some VA-CNTs are still aggregated as exemplified in Figure 5a. The gold nanoparticles can be observed only at one of the ends of these aggregates (Figure 5b and 5c), meaning that metal is deposited on the top surface of the forest and not along the entire length of the CNTs. This fact is easily explained by the technique used for the decoration of VA-CNTs by gold nanoparticles, i.e., magnetron sputtering, technique well-known for surface functionalization. Based on the TEM images, gold nanoparticles exhibit a spherical shape and a mean diameter of about 6 nm. The size of the nanoparticles was mainly controlled by the duration of the process with the given sputtering parameters.

**Figure 6:**
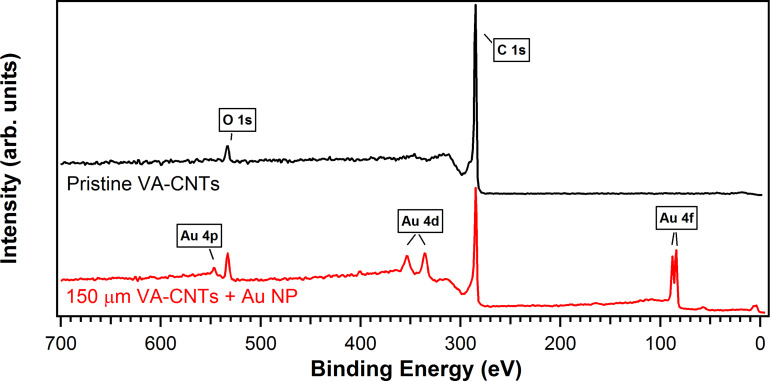
XPS survey spectra for pristine VA-CNTs (top, black line) and 150 µm-long VA-CNTs decorated with 6 nm-diameter gold nanoparticles on top (bottom, red line).

XPS analyses were performed in order to evaluate if chemical changes were induced on the CNTs after the gold decoration. Figure 6 shows the XPS survey spectra of CNTs before and after the deposition of gold nanoparticles. On both spectra, the peaks at 284.3 eV and at 533.0 eV, generated by photoelectrons emitted from the C 1*s* and O 1*s* core levels, respectively, can be observed. The atomic concentration of oxygen at the CNT surface increases from 4.0 to 8.5% after gold decoration. The presence of oxygen is intrinsic to the CNT CVD synthesis and mainly arises from the oxygen adsorbed on their surface, while its increase after the decoration can be due to the generation of defects during the gold evaporation. In addition to these peaks, the spectrum recorded after Au evaporation shows additional structures generated by photoelectrons emitted from Au atoms: the more prominent ones being the 4*f* doublet components at binding energy of 84.0 eV (4*f*_7/2_) and 87.6 eV (4*f*_5/2_), the 4*d* doublet at 335.1 eV (4*d*_5/2_) and 353.2 eV (4*d*_3/2_) and the component 4*p*_3/2_ at 546.3 eV of the 4*p* doublet.

**Figure 7:**
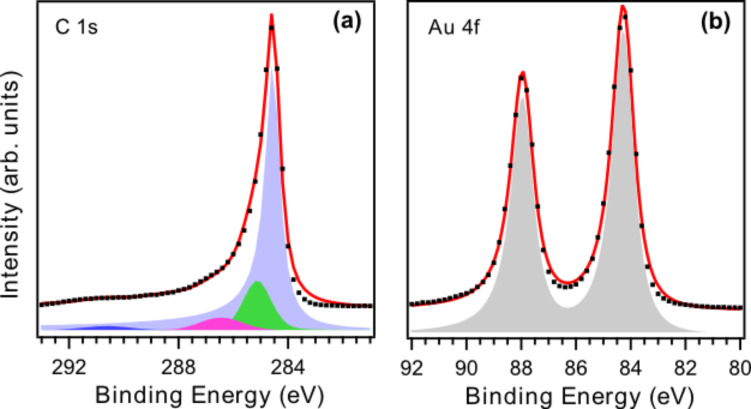
XPS core level spectra of C 1*s* (a) and Au 4*f* (b) recorded on 150 µm-long VA-CNTs decorated with 6 nm-diameter gold nanoparticles on top. A Shirley background has been subtracted, experimental data (black dots) and peaks resulting from a least-square fitting procedure (red lines). Fitting peaks of C 1*s* (a): light blue for sp^2^ carbon, green for sp^3^ amorphous carbon, purple for C-O and dark blue for π-plasmon excitations.

The C 1*s* core level spectrum is the best monitor of the chemical changes onto the CNTs surface. Figure 7a presents the C 1*s* core level recorded on the 150 μm-long VA-CNTs decorated with 6 nm-diameter gold nanoparticles. A Shirley background has been subtracted. The main peak at 284.3 eV is generated by sp^2^ hybridized graphitic carbon atoms located on the walls of the CNTs, it is strongly asymmetric and it has been fitted by a Doniach–Sunjic lineshape with an asymmetry parameter α equal to 0.1. Voigt profiles were used to reproduce the other features observed in the spectrum: the peak at 284.9 eV is associated to photoelectrons emitted from amorphous carbon atoms with sp^3^ bonds, formed during the CNTs synthesis as also confirmed by the defects imaged by HR-TEM (Figure 3) and by the D band measured by Raman (Figure 4); the peak at 290.4 eV corresponds to the electron energy loss peak due to π-plasmon excitations. These three peaks are characteristics of C 1*s* core level from CNTs [28]. The additional small peak (relative area of 5%) at 286.4 eV is due to the presence of oxygen [29]. The low intensity of the components related to defects and the presence of oxygen indicates that the sp^2^ structure is preserved.

Figure 7b presents the Au 4*f* core level spectrum used as a reference to align the binding energies. It can be fitted by a doublet of Doniach–Sunjic lineshapes with an asymmetric parameter of 0.12, the core level shift between 4*f*_7/2_ and 4*f*_5/2_ being 3.68 eV, in agreement with tabulated values [30].

### Gas sensing properties

Sensors based on Au-decorated VA-CNTs with different lengths responded to NO_2_ at room temperature. However, mild heating at 150 °C was needed to help desorb the species from the surface so the baseline resistance could be fully recovered. This is not surprising because a rather strong interaction (chemisorption) between oxygen plasma treated or gold nanoparticle decorated CNTs and NO_2_ has been reported [22,31]. Sensor resistance decreased in the presence of NO_2_ (an oxidizing species), which confirms that the VA-CNT carpets behave as *p*-type semiconductors.

**Figure 8:**
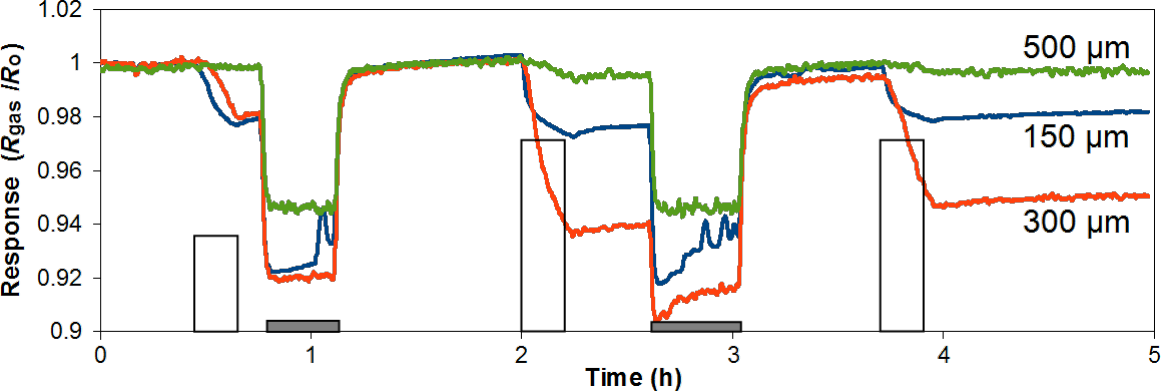
Room temperature detection of NO_2_ for sensors with different CNT lengths. White pulses indicate the exposure to 0.5 ppm, 1 ppm and 1 ppm of nitrogen dioxide (duration was 15 min). Grey bars indicate the periods of heating at 150 °C that help clean the surface of CNT after being exposed to nitrogen dioxide. Heating is not applied after the last exposure cycle and baseline is not regained.

The response to NO_2_ depends on the length of the VA-CNTs (Figure 8). The highest response is obtained for sensors employing 300 µm-long tubes (Table 1). This can be due to the fact that these sensors offer the highest effective surface area (or optimal surface to volume ratio) for interaction with NO_2_. The fact that VA-CNTs form a very compact forest may make difficult for NO_2_ to diffuse and adsorb within the entire thickness of the film in the case of 500 µm-long tubes. Additionally, the chemical sensitization effect of Au nanoparticles sitting on top of the CNT film may be lost beyond a given depth, which would also explain the loss of sensitivity found in 500 µm-thick films. This result could change if the density of the carbon nanotube forest changed (e.g., lower densities would enable a complete Au sensitization or a better diffusion of nitrogen dioxide along the entire film).

**Table 1:** Relative resistance change to NO_2_ depending on the humidity level.

	Relative resistance change to NO_2_ Δ*R*/*R*_0_ (%)			
Length (μm)	0.5 ppm @ dry air	1 ppm @ dry air	0.5 ppm @ 50% R.H.	1 ppm @ 50% R.H.

150	2.2	2.8	3.5	5
300	2	6.2	4	7.5
500	—	<0.5	2	4

The pattern of response to nitrogen dioxide remains basically unchanged when the background humidity level is changed from dry to 50% R.H. However, a general increase in response is observed. These results are in agreement to what was reported by Yao and co-workers [32].

Considering that the active sites are oxygenated defects and Au nanoparticles, a possible reaction pathway for NO_2_ on the surface of CNTs when the humidity level is very low is as follows [31,33]:

[1]
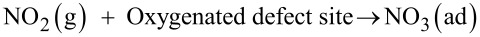


[2]
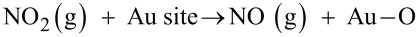


[3]
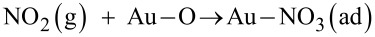


At an intermediate humidity level such as the one tested here (i.e., 50% R.H.), an additional reaction pathway is [34]:

[4]
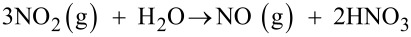


[5]
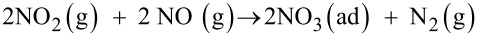


At very low humidity levels, since the amount in weight % of catalyst loading is small, the generation of NO_3_ will remain low and NO_2_ will be the dominating species. However, at higher humidity levels, the HNO_3_ produced according to reaction (4) will form C=O bonds on the surface of CNTs, which will enhance reaction (1). Additionally, reaction (4) also enables reaction (5). As a result, NO_3_ will become the dominating species in the presence of humidity and the overall response is increased [31].

## Conclusion

We have shown that low temperature CVD grown vertical aligned CNTs can be used as active layer for gas sensing. The characterization of the VA-CNTs samples showed that the low temperature growth lead to the formation of defects at the CNT surface however the perfect vertical alignment was obtained. The deposition of gold particles by PVD restricts the formation particles at the sample surface (region near the tips), a slight increase in the oxygen content after the gold deposition was associated with formation of defects.

The CNT length was found to play an important role in the sensitivity for detecting nitrogen dioxide at room temperature when films of VA-CNTs decorated with gold nanoparticles of 6 nm diameter are used as sensors. The sensing test showed that an 'optimal' length of 300 µm for the VA-CNTs maximizes the response towards NO_2_. This fact is explained by the highest effective surface area obtained with 300 µm CNT length for interaction with NO_2_. Upon studying the influence of humidity level from dry to 50% R.H., a global increase in the sensing response was observed for all the sensors regardless of their lengths.
